# Complex yeast–bacteria interactions affect the yield of industrial ethanol fermentation

**DOI:** 10.1038/s41467-021-21844-7

**Published:** 2021-03-08

**Authors:** Felipe Senne de Oliveira Lino, Djordje Bajic, Jean Celestin Charles Vila, Alvaro Sánchez, Morten Otto Alexander Sommer

**Affiliations:** 1 The Novo Nordisk Foundation Center for Biosustainability, Technical University of Denmark, Kongens Lyngby, Denmark; 2 Department of Ecology and Evolutionary Biology, Yale University, New Haven, CT USA; 3 Microbial Sciences Institute, Yale University, West Haven, CT USA

**Keywords:** Industrial microbiology, Applied microbiology, Microbial ecology, Computer modelling

## Abstract

Sugarcane ethanol fermentation represents a simple microbial community dominated by *S. cerevisiae* and co-occurring bacteria with a clearly defined functionality. In this study, we dissect the microbial interactions in sugarcane ethanol fermentation by combinatorically reconstituting every possible combination of species, comprising approximately 80% of the biodiversity in terms of relative abundance. Functional landscape analysis shows that higher-order interactions counterbalance the negative effect of pairwise interactions on ethanol yield. In addition, we find that *Lactobacillus amylovorus* improves the yeast growth rate and ethanol yield by cross-feeding acetaldehyde, as shown by flux balance analysis and laboratory experiments. Our results suggest that *Lactobacillus amylovorus* could be considered a beneficial bacterium with the potential to improve sugarcane ethanol fermentation yields by almost 3%. These data highlight the biotechnological importance of comprehensively studying microbial communities and could be extended to other microbial systems with relevance to human health and the environment.

## Abstract (web summary)

Industrial sugarcane ethanol fermentations are accomplished by a microbial community dominated by *S. cerevisiae* and co-occurring bacteria. Here, the authors investigate how microbial community composition contributes to community function and reveal the role of acetaldehyde in improving yeast growth rate and ethanol production.

## Introduction

Microorganisms live in complex communities that span from simple multicellular aggregates to complex microbiomes composed of thousands of different species^1,2^. Microbial communities are shaped and stabilized by the interactions between their constituent members^3^. These interactions define the composition, dynamics, and functionality of the microbial community^4^. Sugarcane ethanol fermentation with synthetic microbial communities represent a potentially interesting actual microbial community that is tractable for a comprehensive assessment. We have previously shown that these communities have similar compositions across different biorefineries and follow similar community adaptations during the industrial process^5^.

Further understanding of microbial interactions are crucial from microbiological, ecological, and biotechnological perspectives^6^, but progress is hindered by the complexity of most natural communities. Synthetic microbial communities represent important tools for studying microbial interactions^7–9^, as they can be directly manipulated and their responses can be precisely quantified^10,11^.

The microbial community from sugarcane ethanol fermentations is reproducible, and simplified versions can be established in the laboratory^12–14^, providing researchers with a wealth of data regarding fermentation parameters that are directly linked to microbial physiology and community functionality (e.g., ethanol titre, biomass growth, and organic acid titres)^12^. In addition, bioethanol production has both economic and environmental relevance. In Brazil, sugarcane refineries produced >34 billion litres of ethanol in 2019, employing over one million people^15^. During the last 13 years, bioethanol has been responsible for avoiding the release of more than 500 million tons of CO_2_ in the atmosphere, and bioethanol replaced >40% of the gasoline consumed in Brazil^16^.

This industry makes use of the Melle–Boinot fermentation process, which is based on high cell density fed-batch fermentations^17^. The fermentation broth is typically composed of sugarcane molasses diluted with water or sugarcane juice and usually has a sugar concentration of *ca*. 18–22% (w/v basis)^18^. Large fermenters, with volumes normally exceeding 500 thousand litres, are fed fresh broth for ~4–6 h^19^. Selected strains of *Saccharomyces cerevisiae* convert the sugars present in the broth into ethanol, reaching final concentrations of 7–11% (v/v basis), with fermentation yields as high as 92% of the total theoretical yield^17^. The high cell density of yeast cells in these fermentations (~10%, wet weight/volume) allows for very fast fermentations, usually lasting for 6–12 h^17^. After the fermentation is complete, cells are separated from the broth via centrifugation^17^. After centrifugation, these cells are diluted with water and acid-washed for preventive control of contamination for approximately 1 h, after which cells are pumped back into the fermenters for a new batch of fermentation^19^. This yeast biomass can be continuously recycled through the production season, resulting in almost 600 fermentation cycles^19^.

The Melle–Boinot process is operated under non-sterile conditions, which makes contamination commonplace^17,19^. The most common contaminants are lactic acid bacteria, which are able to form stable populations throughout the production season^20–23^. It is considered that such contaminants are mostly detrimental to the production process, as they will compete with yeast for the available nutrients, and their metabolic products are inhibitory towards yeast cells^22^. In this work, we use a combination of flux balance analysis and combinatorial microbial culture to assess the functional landscape of microbial assemblies reflecting the sugarcane ethanol process.

## Results

### Competition shapes the microbial community of sugarcane biorefineries

**Fig. 1:**
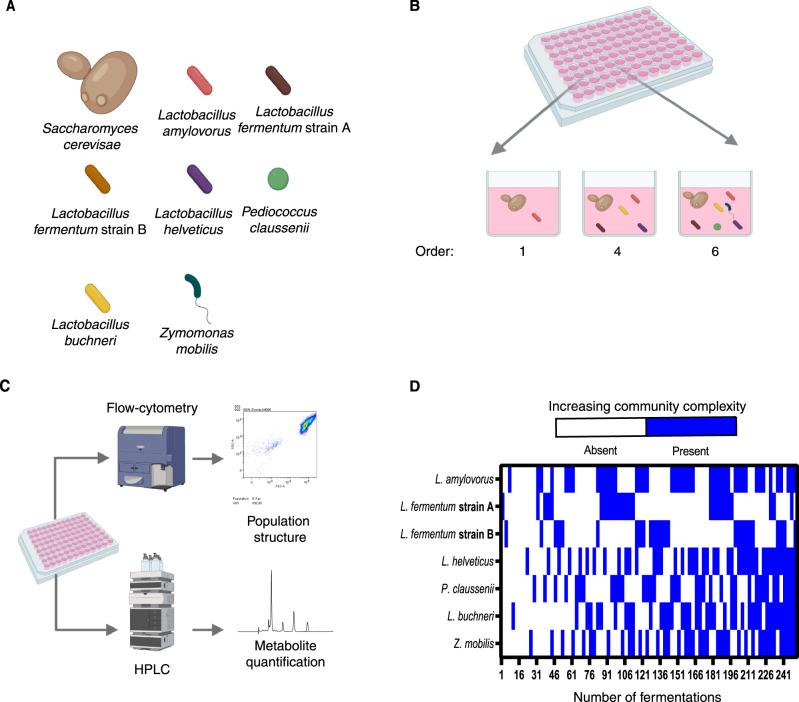
Schematics of the combinatorial assembly experimental setup. **A** Microbial species used in this study. These species make up >80% of the diversity found in fuel ethanol fermentations in relative abundance. **B** Initially, pairwise fermentations between yeast inoculum and all available bacteria were performed. This results in a combinatorial assembly of order 1. In parallel, every pairwise assembly was also mixed with every other bacterial monoculture, resulting in a set of 51 different fermentations or order 2. This was performed for every possible combination of bacterial species and yeast, resulting in 258 fermentations, and a total order of 6 (i.e., a community containing up to six different bacterial species and yeast). The ratio between all bacterial species was kept at 1 for every cultivation, and the yeast:bacteria ratio was 100:1^16^. **C** After fermentation was completed, the community structure was analysed via flow cytometry, and the fermentation metabolites were quantified via HPLC. **D** An overview of the microbial compositions generated via the combinatorial assembly throughout the 258 fermentations. This figure was created by FSOL using Biorender (https://app.biorender.com/).

We sought to develop a simplified yet realistic microbial consortium that could be used as a model system for studying and understanding microbial interactions in industrial sugarcane ethanol biorefineries. To that end, we constructed a synthetic consortium composed of *Lactobacillus amylovorus*, *Lactobacillus fermentum*, *Lactobacillus helveticus, Pediococcus claussenii*, and *Zymomonas mobilis*, in addition to the dominant species *Saccharomyces cerevisiae*, which is responsible for ethanol fermentation (Fig. 1A). These bacterial species represent >80% of the contaminant community found in industrial sugarcane ethanol fermentations^5^.

To unravel how each species and their interactions contribute to the desired performance output (ethanol yield), we combinatorically reconstituted every possible microbial consortium that contained yeast as well as any number (from zero to six) of the bacterial species growing together^8^ (Fig. 1B; Methods). Each synthetic community was inoculated with yeast/bacteria at a ratio of 100:1 to replicate actual industrial conditions^17^. All bacterial species in the inoculum were included at equal proportions. Microbial consortia were incubated for 24 h, after which both cells and media were separated and harvested to analyse community structure (yeast and bacterial population size and relative abundance) via flow cytometry and function (final ethanol yield) via high-pressure liquid chromatography (HPLC) analysis (Fig. 1C, Methods). Three replicates for each condition were run, leading to a total of 258 fermentations (Fig. 1D).

**Fig. 2:**
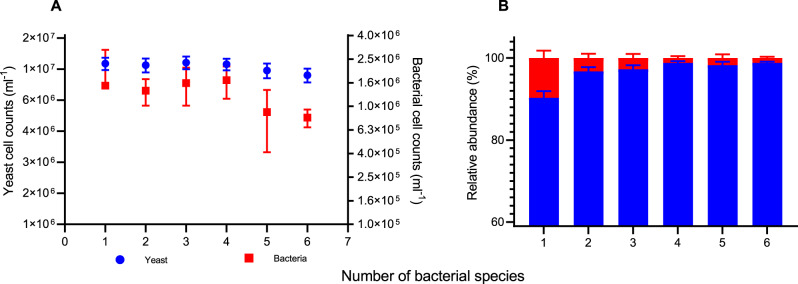
The community structure of yeast and bacteria co-cultivations. **A** Final cell counts of yeast (blue) and bacteria (red) from different community structures. The final yeast cell counts dropped from 1.1 × 10^7^ ± 1 × 10^6^ to *ca*. 9.1 × 10^6^ ± 9.3 × 10^5^ cells mL^−1^ as the number of bacterial species in the community increased (one-way ANOVA, *p* < 0.0001). The final bacterial cell counts dropped from 1.5 × 10^7^ ± 1.5 × 10^6^ to *ca*. 8 × 10^5^ ± 1.4 × 10^5^ cells mL^−1^ (one-way ANOVA, *p* = 0.0002). Symbols and error bars represent the mean ± standard deviation. **B** The relative abundance of yeast and bacterial populations in different community structures. The relative abundance of the yeast population (blue bar) increased from 90.3 ± 1.6% in pairwise cultivations to 98.8 ± 0.3% in the presence of all six different bacterial species (one-way ANOVA, *p* < 0.0001). The bacterial relative abundance (red bar) decreased from 9.7 ± 1.8% initially in pairwise cultivations to 1.2 ± 0.3% in the most complex community tested, which contained yeast and six different bacterial species (one-way ANOVA, *p* < 0.0001). Columns and error bars represent the mean ± standard deviation. For all the results presented in the figure, *n* = 3 independent experiments. Source data are provided as a Source Data file.

The population size of both yeast and bacteria generally decreases in total cell count following the increase in the number of different species added to the consortia, suggesting that competition is a major interaction shaping these communities^24–31^. The yeast population dropped by 16% from *ca*. 1.1 × 10^7^ ± 1 × 10^6^ cells mL^−1^ when grown in monoculture to ~9.1 × 10^6^ ± 9.3 × 10^5^ cells mL^−1^ when grown in co-culture with all six bacterial species. The decline in population size was statistically significant (*p* < 0.0001, one-way analysis of variance (ANOVA); Fig. 2A). The bacterial population also dropped almost 50% when comparing the cell counts when a single species of bacteria was inoculated with yeast to when all six bacterial species were present in the consortium (*p* = 0.0002, one-way ANOVA; Fig. 2A). Both populations were analysed via flow cytometry.

Although both yeast and bacterial populations declined as diversity increased, the relative abundance of yeast grew with increasing diversity (Fig. 2B). Yeast relative abundance shifted from 90.3 ± 1.6% in pairwise consortia to 98.8 ± 0.3% in consortia containing six different bacterial species. In contrast, the relative abundance of the bacterial population fell from 9.7 ± 1.8% from one species to 1.2 ± 0.3% in cultivations where the six species were present. The changes in population structure were both statistically significant (*p* < 0.0001; one-way ANOVA in both cases).

### Higher-order interactions stabilize the performance of yeast and bacterial fermentations

**Fig. 3:**
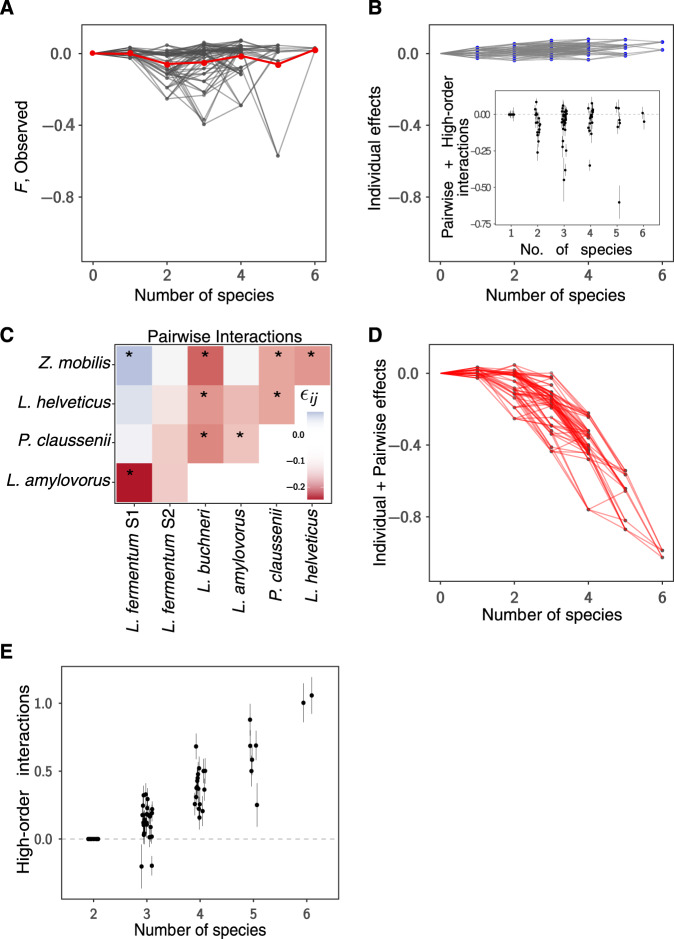
The ecological interactions within the sugarcane biorefinery microbial community. **A** Observed functional landscape. We plotted the measured function for communities of different species. Each line represents the effect of the addition of one species to a community. The red line represents the average of all points for each community size. **B** The expected functional landscape when considering the presence/absence (additive) null model. The inset shows the difference between the measured and predicted functions, plotted against community size, as a way to visualize the fit of the data to the null model. Symbols and error bars represent the mean ± standard deviation. **C** Heat map showing the interactions among pairs of bacterial species (asterisks represent significance taking ± 2SE). These interactions were diverse and mostly detrimental to ethanol yield. The epistasis difference (epsilon) between these species also suggests that such interactions are strain driven. **D** The functional landscape was predicted by adding up single and pairwise effects. This landscape predicts a rapid reduction in function as more species are added. **E** The difference between the prediction of the model in **D** is plotted against community size. For visualization, a jitter was applied to points of different composition but the same community size. The plotted difference effectively measures the influence of high-order interactions. Symbols and error bars represent the mean ± standard deviation. For all the results presented in the figure, *n* = 3 independent experiments. Source data are provided as a Source Data file.

In order to better understand the influence of interactions between different bacterial species on ethanol yield, we studied the functional landscape of our communities, a method used to assess how the function of a community relates to its composition^8^. To that end, we characterized the community function (*F*) as the logarithm of the ratio between the ethanol yield (*Y*) observed when yeast was grown in a mixed culture with any given bacterial consortium (Eq. 2, Methods) and the ethanol yield for yeast in monoculture. In this definition, *F* = 0 means that the bacterial consortium has no effect on ethanol production compared to yeast growing alone. The measured functional landscape shows a non-monotonic trend in which communities with either very low or very high diversity are less detrimental to ethanol yield than communities with intermediate diversity (Fig. 3A).

To quantitatively characterize the interactions in our communities, we propose a null model that the effects of bacterial species on ethanol yield combine additively, such that the impact of adding an additional species to the community is equal to its effect when co-cultured alone with yeast. Such a null model was previously shown to be reasonable under a variety of different assumptions regarding the relation between species abundance and function^8^. In our communities, this null model correctly predicted the function of 36/76 communities (47.4%, Figs. 3A, B). We next attempted to improve the predictions in communities with three or more species by including pairwise interactions in our null model, which can be computed by comparing the one- and two-species co-cultures with yeast (Methods). Given that most of the pairwise interactions were negative (Fig. 3C), our model predicted that function would decrease as a consequence of the addition of new species **(**Fig. 3D). However, this was not the case, and the model including pairwise interactions performed substantially worse than the simple, additive model (Fig. 3E; compared with inset Fig. 3B).

Effectively, the difference between the measured values and the predictions of the model including both single and pairwise effects (Fig. 3E) measures the effect of functional high-order interactions (HOIs) (Methods). As expected, our results indicate that the influence of HOIs greatly increases with the addition of new species to the community. Quantitatively, the observed HOIs not only neutralize the effects of the negative pairwise interactions, in which case we would expect the detrimental effect observed in pairs to be kept equal when adding more species. The HOIs actually remove these detrimental effects, driving the function to become almost equal to that of yeast alone (Fig. 3A). In other words, although the addition of most pairs of bacterial species to the yeast cultures had generally adverse effects on ethanol yield, the addition of a diverse community of contaminants with up to six species had beneficial effects, pushing yields to values close to those found for yeast monocultures. This dissection of the interactions found in this community suggests that HOIs play an important stabilizing role in sugarcane ethanol fermentations.

### *Lactobacillus amylovorus* benefits yeast via acetaldehyde production

**Fig. 4:**
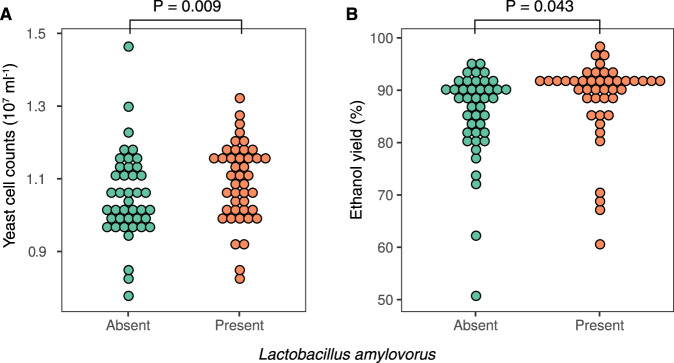
The effect of the presence of *L. amylovorus* on ethanol fermentations. **A** Fermentations performed with *L. amylovorus* (beige circles) in the composition of the microbial community showed higher fermentation yields (3.02 ± 1.06%, *p* = 0.009; one-sided Wilcoxon rank-sum analysis) in comparison with the others lacking this species (green circles). **B** Fermentations with microbial communities containing *L. amylovorus* (beige circles) showed higher yeast cell counts (one-sided Wilcoxon rank-sum analysis, *p* = 0.043) in comparison with the others lacking this species (green circles). For all the results presented in this figure, *n* = 3 independent experiments. Source data are provided as a Source Data file.

To pinpoint specific taxa that influenced community function, we performed correlation analysis of the community composition and ethanol yield. The most significant correlations between measured parameters were weak (Supplementary Table 1), suggesting that different species have similar impacts in the community, e.g., redundancy between species^32^. However, the presence of one of the species (*L. amylovorus*) in the consortia was significantly correlated with higher fermentation yields (Wilcoxon rank-sum analysis; *p* = 0.009). Indeed, fermentations containing *L. amylovorus* had 3.02 ± 1.06% higher ethanol yields compared with those lacking this species. (Fig. 4A). In addition to its impact on the yeast population and ethanol yield, the presence of *L. amylovorus* was also positively correlated with yeast growth (Wilcoxon rank-sum analysis; *p* = 0.043; Fig. 4B).

The increase in yeast population and ethanol yield might be partly explained by the possible antagonistic effect of this species against other bacteria in the fermentation environment^33–37^. However, considering that this statistical analysis indicates only when this species is present, independent of the community’s configuration, we decided to further analyse whether *L. amylovorus* may have a positive effect on yeast metabolism. One mechanism for such a positive impact might be through a cross-feeding interaction. To investigate the possibility of cross feeding between *S. cerevisiae* and *L. amylovorus*, we simulated the growth of *S. cerevisiae* via flux balance analysis (FBA), both in the presence or absence of *L. amylovorus-*secreted by-products. In this simulation, the growth of both species was simulated separately in a defined environment. The secreted by-products and the outer fluxes from *L. amylovorus* growth were used to complement the media composition used in the *S. cerevisiae* growth simulations (Methods).

The yeast model^38^ lacked the capacity to consume some metabolites produced from *L. amylovorus*. These metabolites, present in minute concentrations compared with the main metabolites, were not considered to simplify further analyses. The simulation predicted that yeast would consume the acetaldehyde produced from *L. amylovorus*, resulting in higher biomass and ethanol yields.

**Fig. 5:**
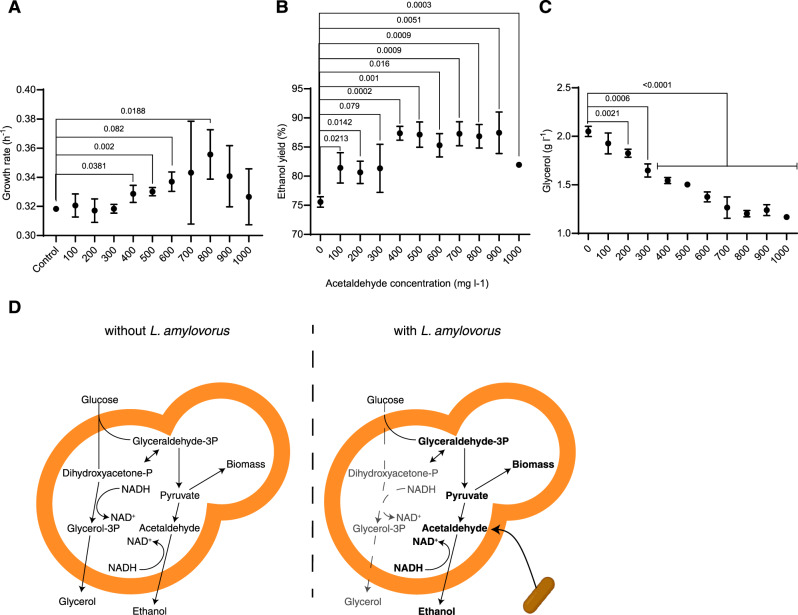
The effect of different concentrations of acetaldehyde on yeast physiology and proposed biochemical mechanism. **A** Comparison between the growth rates (h^−1^) of the yeast population in the presence of different concentrations of acetaldehyde. An optimal concentration of acetaldehyde (between 400 and 800 mg L^−1^) results in growth rates higher than the control (0). Values over lines represent significant *p* values from one-sided unpaired *t* tests. Symbols and error bars represent the mean ± standard deviation. **B** Ethanol yield (%) of yeast fermentations in the presence of different concentrations of acetaldehyde. The highest values of ethanol yield were achieved when the acetaldehyde concentration fell within a range of 400–900 mg L^−1^. Values over lines represent significant *p* values from one-sided unpaired *t* tests. Symbols and error bars represent the mean ± standard deviation. **C** Glycerol production by yeast cells decreased linearly, following increased acetaldehyde concentrations in the fermentations. This decrease was not followed by other measured parameters, such as biomass production, growth rate, and ethanol yield (Fig. 4), suggesting a certain uncoupling of glycerol production and growth in the presence of acetaldehyde. Values over lines represent significant *p* values from one-sided unpaired *t* tests. Symbols and error bars represent the mean ± standard deviation. **D** Proposed mechanism of the metabolic interaction between *L. amylovorus* and *S. cerevisiae*. A representation of yeast growing alone (without *L. amylovorus*) is shown in the left figure, and a representation of yeast growing in the presence of *L. amylovorus* (with *L. amylovorus*) is shown in the right figure. During growth, yeast produces glycerol mainly to balance its redox potential by oxidizing NADH into NAD^+^ via the reduction of dihydroxyacetone-P into glycerol-P. This step is necessary owing to the accumulation of NADH during fermentation^40^. Glycerol is then passively diffused to the extracellular environment, similar to ethanol. Glycerol is also important to maintain the osmo-tolerance of yeast cells, and its production can be induced by high salt and organic acid concentrations^53^. *L. amylovorus* produces an extra pool of acetaldehyde, which can be reduced into ethanol via the ADH1 (alcohol dehydrogenase 1) enzyme in yeast. This extra pool of acetaldehyde pushes the equilibrium towards ethanol production, and most of the NADH will be oxidized in this step. This will result in lower glycerol production (faint arrows and words) and higher ethanol production (bold arrows and words). In addition, this extra acetaldehyde pool can lead to pyruvate accumulation, resulting in higher biomass production (bold arrows and words). Several biochemical steps were suppressed for the sake of clarity. For all the results presented in the figure, *n* = 3 independent experiments. This figure was created by FSOL. Source data underlying Fig. 5a–c are provided as a Source Data file.

In order to validate these findings, we compared the growth and fermentation performance of *S. cerevisiae* in the presence of acetaldehyde with concentrations between 0 and 1000 mg L^−1^ ^39^. The results suggest that acetaldehyde was able to stimulate the growth rate (Fig. 5A) of *S. cerevisiae*, in a range from 400 to 800 mg L^−1^, as well as the ethanol yield, resulting in an increase of >10% (Fig. 5B). The observed results also suggest that the stimulation of yeast growth and ethanol production had an optimum value in a particular acetaldehyde concentration range. Tested concentrations indicate that higher concentrations of acetaldehyde (from 700 to 1000 mg L^−1^) become inhibitory for yeast growth rate (Fig. 5B) and biomass production (Supplementary Fig. 1) when compared with more intermediate concentrations.

Indeed, acetaldehyde is a toxic compound to yeast, and its stimulatory effects are concentration-dependent and can become inhibitory above certain threshold values under specific physiological conditions^39,40^. The redox potential of yeast cells is balanced by two main reactions during fermentations: via the reduction of dihydroxyacetone phosphate to glycerol-3-phosphate (G3P), a reaction catalysed by cytosolic NAD^+^-dependent G3P dehydrogenase, or by the reduction of acetaldehyde into ethanol via alcohol dehydrogenase 1^41^. This is in line with the FBA simulations, which predicted that 97% of the acetaldehyde flux would go towards ethanol production via alcohol dehydrogenase reduction. Therefore, the provision of a higher acetaldehyde pool as an alternative electron acceptor could allow yeast cells to rebalance their NADH/NAD pools solely via ethanol production, allowing cells to distribute more carbon towards pyruvate and biomass production. This carbon rerouting would result in higher ethanol and biomass titres, with the expense of glycerol production. To test this hypothesis, we quantified the glycerol produced by yeast exposed to different acetaldehyde concentrations. As expected, we observed a continuous reduction in glycerol titres following acetaldehyde concentration (Fig. 5C).

In light of these results, it was necessary to identify which species might contribute to the potential acetaldehyde pool in fermentation. When analysing the supernatants from bacterial cultivations, it is possible to identify *L. amylovorus* as the major aldehyde producer, corroborating the previous observations. The concentration of acetaldehyde in *L. amylovorus* supernatant was *ca*. 460 mg L^−1^ (Supplementary Table 2). According to our experimental analysis, this value could provide both higher ethanol yield and growth rate for the yeast population (Figs. 5A and 5B).

Based on these results, we hypothesize that under the given fermentation environment, *L. amylovorus* secretes acetaldehyde, which is readily assimilated by yeast cells to balance the NAD/NADH cytosolic pool and reduced to ethanol. This implies that lower glycerol and higher yeast biomass and ethanol titres are produced, further increasing the ethanol titre (Fig. 5D).

### Cross feeding between yeast and *Lactobacillus amylovorus* improves the performance of sugarcane molasses fermentations

In order to corroborate the effect of *L. amylovorus* metabolites in yeast fermentations we have performed cross-feeding experiments between *S. cerevisiae* and *L. amylovorus* (Supplementary Table 3). We have compared the fermentation performance of small-scale yeast cultivations performed using fresh undiluted synthetic molasses media^13^ or in media diluted with either water or the supernatant of *L. amylovorus* cultivations. Results suggest that yeast cultivations in media diluted with *L. amylovorus* supernatant are able to achieve similar ethanol titres than cultivations performed in fresh media (Supplementary Table 3, Supplementary Fig. 2).

Such ethanol titres can be partly explained by the residual sugars present in the *L. amylovorus* supernatant, but the total ethanol produced in these conditions surpasses the total available sugars (Supplementary Fig. 3). In fact, the amount of extra ethanol produced in these conditions (i.e., ~12% of total ethanol produced) is in similar range to the acetaldehyde concentration in this supernatant (Supplementary Table 4).

We have also investigated the effects of organic acids in yeast metabolism. Even when grown under high concentrations of both lactic and acetic acids (44.4 and 33.3 mM, respectively) yeast was still able to grow, reaching lower final biomass and ethanol titres (Supplementary Table 3).

With this additional indication of metabolic interaction between both species, we investigated the effect of *L. amylovorus* addition on yeast-based sugarcane molasses fermentations. For such, we have simulated, as far as possible, the industrial sugarcane ethanol production process in laboratory scale^12^, and compared the main fermentation parameters in the presence or absence of different concentrations of *L. amylovorus*.

**Fig. 6:**
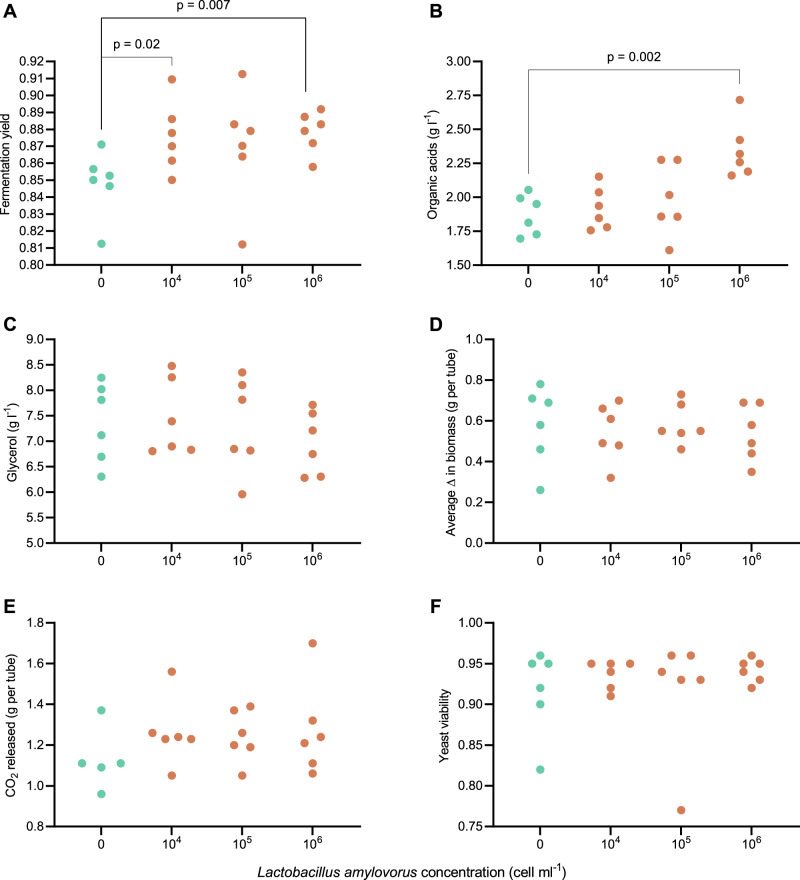
The effect of *Lactobacillus amylovorus* on sugarcane molasses ethanol fermentations. **A** Comparison between ethanol yield values from yeast-based fermentations of synthetic sugarcane molasses inoculated with different concentrations of *L. amylovorus* cells. An increase in ethanol yield is observed following higher bacterial cell concentrations. Values over lines represent significant *p* values from bootstrapping. Beige circles: no *L. amylovorus* added in the fermentation. Green circles: *L. amylovorus* added in the fermentation. **B** The acidity titres (g organic acids L^−1^) from fermentations inoculated with different concentrations of *L. amylovorus* cells. The titres increase following the higher bacterial cell concentrations, suggesting that most of this acidity increase comes from bacterial metabolism. Values over lines represent significant *p* values from bootstrapping. Beige circles: no *L. amylovorus* added in the fermentation. Green circles: *L. amylovorus* added in the fermentation. **C** Glycerol titres from fermentations inoculated with different concentrations of *L. amylovorus* cells. Fermentations inoculated with higher bacterial concentrations present lower glycerol titres, when compared with control conditions. Beige circles: no *L. amylovorus* added in the fermentation. Green circles: *L. amylovorus* added in the fermentation. **D** Average increase in yeast biomass after each fermentation cycle, from fermentations inoculated with different concentrations of *L. amylovorus* cells. After a fermentation cycle is over, yeast biomass is separated from the fermented broth via centrifugation and weighted. Fermentations inoculated with higher bacterial cells presented lower yeast biomass increases, albeit not significantly different from control conditions. Beige circles: no *L. amylovorus* added in the fermentation. Green circles: *L. amylovorus* added in the fermentation. **E** Ethanol productivity (g ethanol h^−1^) can be inferred via CO_2_ production during alcoholic fermentations, by weighting the fermentation tubes. Fermentations inoculated with higher bacterial cell concentrations presented higher productivity values, although not significantly different from control conditions. Beige circles: no *L. amylovorus* added in the fermentation. Green circles: *L. amylovorus* added in the fermentation. **F** Yeast viability from fermentations inoculated with difference concentrations of *L. amylovorus* cells. No difference can be observed among all treatments. Beige circles: no *L. amylovorus* added in the fermentation. Green circles: *L. amylovorus* added in the fermentation. For all the results presented in this figure, *n* = 3 independent experiments. Source data are provided as a Source Data file.

The addition of *L. amylovorus* shows significant changes in fermentation yield, following the concentration of the bacterial population, resulting in an increase of almost 3% on ethanol yield (Fig. 6A). This increase is followed by higher acidity titres (i.e., total organic acids concentration), probably related to bacterial metabolism (Fig. 6B).

Differences in the final titres of glycerol, and changes in yeast biomass production after each fermentation cycle were also observed, with lower values following higher *L. amylovorus* concentrations, albeit without significant differences when compared with control conditions (Fig. 6C and Fig. 6D, respectively). Ethanol productivity of the fermentations can be inferred by calculating the CO_2_ production rate^12^. The data suggest higher productivity titres when high *L. amylovorus* concentrations are present in the fermentation (Fig. 6E). No significant differences were observed on yeast viability values (Fig. 6F). After the fermentation was ceased, the residual acetaldehyde from centrifuged broths was quantified. No significant differences could be observed between the treatments, suggesting that the acetaldehyde that was produced by the bacterial cells was readily consumed by the yeast population, being further reduced to ethanol (Supplementary Table 5).

## Discussion

In this study, we show how synthetic microbial communities and functional landscape analysis can help resolve the interactions found in actual microbial communities^6,7,9,42^. We demonstrate that bacterial competition is a major microbial interaction found in such communities and that higher-order interactions have a key role in buffering deleterious pairwise functional interactions between bacteria and yeast. These findings are in accordance with previous studies showing that competition is the major interaction found in microbial communities^26^ and that higher-order interactions are common forces involved in shaping the functionality of microbial communities^8,43^. Our findings also show that contamination control methods should be more precise^43^ and should discriminate among different species of bacteria^44^. Our analysis identified a specific bacterial contaminant-*L. amylovorus*-whose presence was positively associated with higher ethanol yields. We found that this may be caused by two different mechanisms^45,46^.

FBA simulation of yeast growing in the presence of the by-products secreted by *L. amylovorus* identified a second potential mechanism that may also lead to increased ethanol yield, and this mechanism was later validated via laboratory experiments. Such a mechanism is based on the reduction of acetaldehyde produced by bacteria into ethanol by yeast to balance the cytosolic redox potential^39,41^. This results in lower glycerol production and higher ethanol yields by the yeast.

Although *L. amylovorus* has a positive effect on yeast ethanol production, we found that this effect is contingent on the additional presence of other taxa in the consortia. Our findings related to the stabilizing effect of HOIs indicate that biodiversity in the bacterial community is positively associated with the ethanol function of our consortia and that the beneficial effect of *L. amylovorus* is exhibited when in co-culture with other bacterial contaminants. This finding opens the way to identifying specific multispecies consortia that may optimally promote bioethanol production.

Maintaining a viable population of *L. amylovorus* seems advantageous for industrial processes. Simulated sugarcane ethanol fermentations inoculated with high concentrations of *L. amylovorus* presented higher fermentation yields compared with control conditions, resulting in almost a 3% yield increase. The observed increase in acidity titres was not detrimental to the yeast population, as other fermentation parameters (like viability) had similar values to control conditions. Moreover, yeast is capable of providing a niche to certain lactobacilli via its nitrogen metabolism^47^. This could result in a mutualistic interaction between these two species, benefiting an industrial process that still regards and responds to bacterial contaminants as being solely detrimental^48^.

In summary, our results suggest that multispecies consortia containing *L. amylovorus* could be considered a class of industrial probiotics or, in other words, a beneficial bacterium to industrial ethanol fermentations. Our findings show that adding these consortia to a fermenter during industrial ethanol fermentations would benefit the yeast population and the overall industrial process performance. We hypothesize that this effect could potentially stem both from competition towards other bacteria^44^, through stimulation of the yeast central carbon metabolism^47^, or both.

## Methods

### Chemicals

Unless stated otherwise, all chemicals and reagents used were of analytical grade and were purchased from Sigma-Aldrich (St. Louis, MO, USA).

### Strains used in laboratory experiments

*Saccharomyces cerevisiae* strain PE-2 was kindly provided by professor Thiago Olitta Basso. Strains of *Lactobacillus amylovorus* and *Lactobacillus fermentum* were isolated from stored industrial samples. Strains of *Pediococcus claussenii*, *Lactobacillus helveticus, Lactobacillus buchneri*, and *Zymomonas mobilis* were purchased from ATCC (Manassas, VA, USA).

### Isolation and maintenance of industrial strains

For strain isolation, a previously introduced protocol was used^20^. In brief, industrial samples were serially diluted in sterile PBS and plated on de Man Rogosa Sharpe (MRS) agar medium plates containing cycloheximide (0.1% v v^−1^) to inhibit yeast growth. Plates were incubated at either 30 °C or 37 °C statically. A loopful of an isolated colony was grown in liquid MRS under the same conditions and stored at −80 °C. Yeast strains were cultured in yeast potato dextrose (YPD) medium at 30 °C. Lactobacilli were cultured in MRS medium, either at 30 °C or 37 °C, and *Zymomonas mobilis* was cultured in trypsin soy broth (TSB) medium, at 30 °C. All cultivations for inoculum preparation were performed statically, ca. 5 mL volume.

### Fermentation experiments and combinatorial assembly

Fermentations were performed in 96 deep-well plates with either pairwise cultivations (yeast:bacteria at a ratio of 100:1)^9^ or standalone yeast or bacteria co-cultivations. A semi-synthetic medium that is able to simulate sugarcane molasses-based medium (SM) was used^13^. In brief, all strains were cultured in their optimal media and conditions for up to 48 h. After that, the biomass was calculated via optical density (OD; 600 nm wavelength). All cells were pelleted by centrifugation (3400 × *g*, 4 °C, 15 min) and washed twice with sterile PBS. Subsequently, cells were diluted in SM diluted in sterile Milli-Q H_2_O (10×, final sugar concentration of 18 g L^−1^) for an OD value of 1.0. Strains were later diluted in fresh SM medium in specific wells in the 96 deep-well plate to a final OD value of 0.1.

All co-cultivations were performed statically overnight at 30 °C in ca. 1 mL of medium, in triplicate. Combinatorially assembled communities of the six bacterial species were prepared by inoculating the same volume from each bacterial seed culture into 96 deep-well plates (VWR) containing 500 µL of 10× diluted SM. The final yeast and bacteria ratio (OD) was maintained at 100. This means that the final cell count of each individual bacterial population decreased as the number of species in the community increased.

The main metabolites from fermentation (i.e., glycerol, ethanol, lactic, and acetic acid) were quantified via HPLC (UltiMate 3000, Thermo-Fischer Scientific, Waltham, MA, USA). The metabolites were differentiated with an Aminex HPX-87H ion exclusion column (Bio-Rad, Hercules, California, USA), being isocratically eluted at 60 °C, with a flow rate of 0.6 mL min^−1^, using a 5 mM sulfuric acid solution as the mobile phase. The detection was performed refractrometrically.

Ethanol yield was calculated as follows:

1 $${\mathrm{Ethanol}}\;{\mathrm{yield}} = \frac{{\left( {{\mathrm{EtOHobs}} \,\times 100} \right)}}{{{\mathrm{EtOHtheor}}}}$$

EtOH_*obs*_: the observed ethanol titre for each sample. EtOH_theor_: the maximum theoretical ethanol titre for each sample. The sugar titre from the broth solution was multiplied with the stoichiometric conversion factor for ethanol production (i.e., 0.5111)^12^.

Community composition was resolved via flow cytometry (BD LSRFortessa™, BD Biosciences, Franklin Lakes, New Jersey, USA). A sample from each well (10 µL) was taken after overnight cultivation, transferred to a new microplate and diluted in 190 µL of PBS buffer (pH 7.4). Yeast and bacterial populations were resolved via front and side scatter comparison (SSC versus FSC). An example of the gating strategy applied can be seen in Supplementary Fig. 4.

### Functional landscape analysis

To study the structure-function landscape of our communities, we expressed the function (*F*) of interest (ethanol yield) as shown below:

2 $$F = lg\left( {\mathrm{Yield}}_{{\mathrm{Yeast}} + \frac{{\mathrm{Community}}}{{\mathrm{Yield}}_{{\mathrm{Yeast}}}}} \right)$$

Community function (*F*) is expressed as total ethanol yield from the community. Here, *F* is defined as the *lg* value of the ethanol yield of standalone yeast fermentations (Yield_yeast_) and the sum of the ethanol yield from different community structures (Community) divided by yeast standalone fermentation values. The ethanol yield ratio was expressed in a logarithmic manner, allowing us to use an additive null model to quantify the interactions among our bacterial species.

Using this model and following the rationale presented elsewhere^8^, we were able to decompose the function of every community, *F*_C_, into single, pairwise and higher-order effects:

3 $$F_{\mathrm{C}} = \sum _{\mathrm{i}}\quad F_{\mathrm{i}} + \sum _{\mathrm{i \ne j}}\quad \in _{\mathrm{ij}} + H_{\mathrm{C}}$$

Community model integrating the effects of single, pairwise and higher-order interactions. *F*_C_ is the community function, *F*_i_ is the single effects functions, *ϵ*_ij_ is the effect of pairwise interactions between species (i and j) and *H*_C_ is the higher-order effects.

The single effects function *F*_i_ can be computed simply as the function of co-cultures of single species with yeast. Pairwise interactions can be estimated as the function of co-cultures of pairs of species with yeast that are not explained by single effects:

4 $${\it{ \in }}_{\mathrm{ij}} = F_{\mathrm{ij}} - F_{\mathrm{i}} - F_{\mathrm{J}}$$

Calculation of pairwise effects (*∈*_ij_), where the function values between yeast and different bacterial species in monoculture (*F*_i_ and *F*_J_) are subtracted from the function value of co-culture pairs (*F*_ij_).

Following the same logic, in communities with three or more members, higher-order interactions can be measured as a function that is unexplained by the sum of single and pairwise effects. By truncating Eq. 2, we can compute the expected structure-function landscape for each community when considering only additive effects (Fig. 2C) or a landscape that takes both single and pairwise effects into consideration (Fig. 2D).

### Construction of metabolic models

Genome-scale metabolic models from the used bacteria were constructed using the automated tool CarveMe^49^. Reference genomes used to construct the models of the following bacteria were downloaded from the NCBI database: *L. amylovorus* 30SC; *L. fermentum* IFO 3956; *L. helveticus* CAUH18; *P. claussenii* ATCC BAA 344; *L. buchneri* CD034; and *Z. mobilis* ATCC 10988.

All models were gapfilled to allow anaerobic growth in minimal medium (M9). The command line used was ‘carve genome.faa --gapfill M9[-O2] -u grampos -o model.xml.gz’, where ‘genome.faa’ stands for the annotated genome of each of the selected microorganisms, and model.xml.gz is the final file name. For *Z. mobilis*, the universal gram-negative model gramneg was used for top–down reconstruction. Prior to any analysis, the models were used to simulate growth in defined conditions (see ‘Flux balance analysis’ section) to corroborate the quality of the model. For *S. cerevisiae*, the model used was the extensively curated iMM904^38^.

### Flux balance analysis

Flux balance analysis simulations were performed using COBRApy^50^. In brief, a simple environment simulating a defined minimal medium was created. In order to sustain yeast growth, the minimal medium allowed the fluxes of NH_4_, SO_4_, Pi, H_2_O, K, Na, and CO_2_ to be unconstrained. The environment was also composed of ergosterol, zymosterol, palmitoleate (C16:1), stearate (C18:20), oleate (C18:1), and linoleate (C18:2), which were freely exchanged (lower bound = −1000). The lower bound of the exchange reaction for oxygen was set to 0 (anaerobic environment), and the carbon sources (glucose, fructose and sucrose) were each set to −10.

The growth of *Lactobacillus amylovorus* and *Saccharomyces cerevisiae* was simulated separately in this environment. To simulate the cross-feeding between both species, the secreted metabolites from *L. amylovorus* were included in the yeast environment, and their exchange fluxes were constrained based on the out fluxes from *L. amylovorus*^51^. Any metabolite for which the yeast model lacked the capacity to assimilate was not considered in this simulation.

### Acetaldehyde quantification

The different bacterial species were grown overnight at 30 °C in 10× diluted SM. After this, the acetaldehyde concentration in the fermented media was measured enzymatically using the Acetaldehyde Assay Kit (K-ACHYD—Megazyme, Bray, Ireland), following the manufacturer’s instructions.

### Analysis of the effect of acetaldehyde on yeast physiology

Acetaldehyde was added to fresh Milli-Q-diluted SM at different concentrations^39^, ranging from 100 to 1000 mg L^−1^, with 100 mg L^−1^ increments. All cultivations had an initial OD of 0.1. The rate of yeast growth was analysed in the presence of different acetaldehyde concentrations at 30 °C under agitation (double orbital, fast mode) in Synergy H1 plate readers (Biotek Instruments, Inc. Winooski, VT, USA). The OD was measured every 15 minutes for 24 h. The growth rate was later calculated using the R package growthcurver^52^. Biomass production and ethanol yield were measured from static fermentations in 10× diluted SM. Fresh medium (5 mL) was inoculated with yeast cells grown overnight in YPD medium. All fermentations were performed statically at 30 °C for 24 h. After fermentation, a sample was taken, and the final OD value was measured. The remaining volume was centrifuged, and an aliquot of 1 mL was saved for HPLC analysis (see section Fermentation experiments and combinatorial assembly).

### Cross-feeding experiments between *L. amylovorus* and *S. cerevisiae*

Both strains were cultured in their optimal media and conditions for up to 48 h (YPD for *S. cerevisiae*, MRS for *L. amylovorus*), and then an aliquot was transferred to fresh Milli-Q-diluted SM. After that, the biomass was calculated via optical density (OD; 600 nm wavelength). All cells were pelleted by centrifugation (3400 × *g*, 4 °C, 15 min) and the supernatant was saved for further use. An aliquot was stored at −20°C for further metabolite quantification (see sections Fermentation experiments and combinatorial assembly and acetaldehyde quantification). Yeast cells were washed twice with sterile PBS, and subsequently diluted in different SM based media: fresh sterile Milli-Q H_2_O diluted SM (10×, final sugar concentration of 18 g L^−1^); fresh sterile Milli-Q H_2_O 2× diluted SM (20×, final sugar concentration of 9 g L^−1^); Milli-Q H_2_O diluted SM mix with *L. amylovorus* supernatant (1:1 v v^−1^); and Milli-Q H_2_O diluted SM mix with organic acids (lactic and acetic acid) solution. The final concentrations of the organic acids were 44.4 and 33.3 mM, for lactic and acetic acid, respectively. The initial OD value was set to 0.1. All cultivations were performed statically overnight at 30 °C in ca. 1 mL of medium, in triplicate. After this period, an aliquot was taken for biomass measurement via OD, and after cell separation via centrifugation, the main metabolites were quantified via HPLC analysis. Fermentation yield was calculated as previously stated (see section Fermentation experiments and combinatorial assembly).

### Industrial sugarcane ethanol fermentations simulations

The fermentation setup employed was adapted from elsewhere^12,13^. In brief, pre-inocula from *S. cerevisiae* and *L. amylovorus* were grown in adequate media for 48 h (see Isolation and maintenance of industrial strains). After this period, both strains were transferred to was inoculated in propagation medium (10° Brix molasses medium, enriched with yeast extract 5 g L^−1^) and was fed daily with fresh media, up to a final volume of 4 L for *S. cerevisiae* and 1 L for *L. amylovorus*, over the course of 4 days^12,13^. In all, 50-mL centrifuge tubes were employed to perform the fermentations. During the first cycle, the cells from the propagation culture were added to each tube in an amount corresponding to 10% (w v^−1^) of the final volume. The cells were fed with undiluted SM (with a final sugar concentration of ~180 g L^−1^) and incubated during 5 h at 30 °C without agitation, and left at room temperature overnight. In the next day, the cells were separated from the fermentation broth via centrifugation (3400 × *g* for 10 min at 4 °C), and broth from the previous cycle was added (70% wet weight w w^−1^), with the intent to simulate the industrial centrifuge efficiency. Whenever necessary, the yeast biomass was adjusted by collecting the excess form each tube with a clean spatula. The cells were further diluted and re-suspended in tap water (30% wet weight w w^−1^) before the addition of 1 M sulphuric acid to a final pH of ~2.5 for 1 h at room temperature, in order to simulate the acid washing of yeast cells. After the acid washing, a new fed-batch fermentation, with fresh must, was initiated, restarting the process. Prior to adding new must, an aliquot of *L. amylovorus* was centrifuged from the stock propagation medium, and the biomass was washed 2× with PBS. The cell concentration (in cells mL^−1^) in the stock propagation medium was calculated via flow cytometry (see section fermentation experiments and combinatorial assembly). The final concentrations of *L. amylovorus* in the fermentations were calculated taking in consideration the final mass of each tube (i.e., considering the yeast biomass, added broth, added water and expected mass of molasses to be added). The *L. amylovorus* concentrations used were: 10^4^, 10^5^, and 10^6^ cells mL^−1^. This pellet was then re-suspended in fresh SM media and used to feed proper tubes. The ethanol produced was calculated as the mass balance difference between the ethanol content from the end of each fermentation cycle (accounting ethanol from the cell-free centrifuged broth plus the pelleted yeast biomass) and the ethanol present in the inoculum (returned broth and the pelleted yeast biomass from the previous cycle)^12,13^. The ethanol yield was calculated based on the total sugar supplied, as shown below^12,13^:

5 $${\mathrm{Ethanol}}\;{\mathrm{yield}} = K\;x\;\left\{ {\left( {{\mathrm{Vw}} + 0.7\;x\;P} \right)\;x\;{\mathrm{ET}} - \left( {{\mathrm{Vrw}} + 0.7\;x\;P{\mathrm{p}}} \right)\;x\;{\mathrm{ETp}}} \right.$$

6 $$K = \frac{{1000}}{{64.75\;x\;{\mathrm{Vs}}\;x\;TRS}}$$

Vw is the volume (mL) of centrifuged broth; *P* is the pelleted yeast biomass (g); ET is the ethanol titre in centrifuged broth (%v v^−1^); Vrw is the volume of returned broth from the previous cycle; *P*p is the pelleted yeast biomass from the previous cycle (inoculum); ETp is the ethanol titre (%v v^−1^) in centrifuged broth from the previous cycle (inoculum); Vs is the volume of substrate (mL); and TRS is the total reducing sugar of substrate (g 100 mL^−1^). Conversion factor of 64.75 mL_ethanol_ 100 g TRS^−1^, equivalent to 51.11 g_ethanol_ 100 gTRS^−1^.

The ethanol productivity was indirectly measured via the CO_2_ production and emission, by weighting the tubes hourly^12^. The viability was measured via flow cytometry (see section Fermentation experiments and combinatorial assembly). The carbohydrate titre and composition from the fermentation media was measured via HPLC (see section Fermentation experiments and combinatorial assembly), with minor adaptations. The oven temperature was set to 30 °C in order to avoid the hydrolysis of sucrose. The concentration of fermentation metabolites was determined by HPLC analysis (see section Fermentation experiments and combinatorial assembly). This experiment was repeated throughout three different fermentation cycles. The acetaldehyde concentration was analysed via enzymatic kit (see section Acetaldehyde quantification) *L. amylovorus* addition started on the second cycle. This experiment was performed in triplicate.

### Statistical analyses

All the statistical analyses mentioned in this study were performed using either GraphPad Prism 9 or R software.

### Reporting summary

Further information on research design is available in the Nature Research Reporting Summary linked to this article.

## Supplementary information

Supplementary Information

Peer Review File

Reporting Summary

## Data Availability

Data supporting the findings of this work are available within the paper and its Supplementary Information files. A reporting summary for this article is available as a Supplementary Information file. The data sets and materials generated and analysed during the current study are available from the corresponding author upon request. The yeast model used was the iMM904 (http://bigg.ucsd.edu/models/iMM904), which is generated from *S. cerevisiae* strain S288 and deposited in NCBI under accession GCF_000146045.2 [https://www.ncbi.nlm.nih.gov/assembly/GCF_000146045.2/]. The *L. amylovorus* strain 30SC model was created from genome GCA_000191545.1 [https://www.ncbi.nlm.nih.gov/assembly/GCA_000191545.1]. Source data are provided with this paper.

## Source data

Source Data

## Code availability

The codes used for performing the flux balance analyses and the metabolic models of *S. cerevisiae* and *L. amylovorus* are available at Github [https://github.com/djbajic/sugar_cane].
