# Supplementary material for: Complex yeast–bacteria interactions affect the yield of industrial ethanol fermentation
Source: Nat Commun. 2021 Mar 8;12:1498. doi: 10.1038/s41467-021-21844-7 (PMC7940389; doi:10.1038/s41467-021-21844-7)
Supplement: Supplementary file 1 — Supplementary Information [file 41467_2021_21844_MOESM1_ESM.pdf]

# Complex yeast-bacteria interactions affect the yield of industrial ethanol fermentation

Lino *et al.*

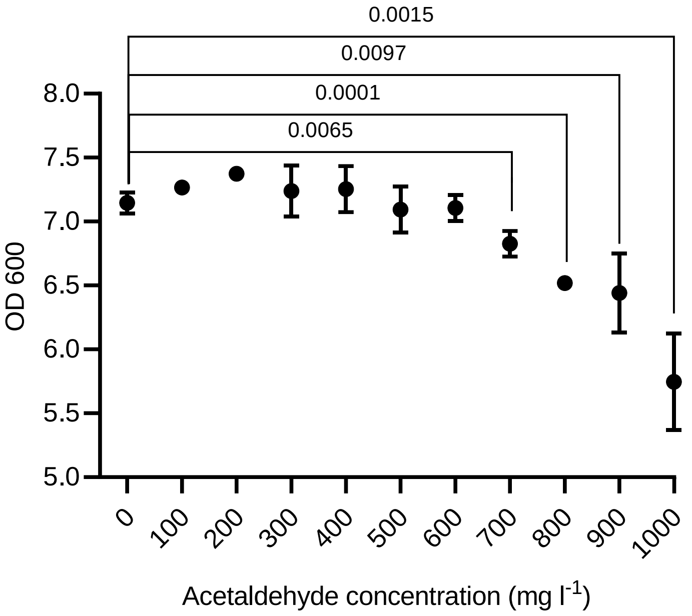


**Supplementary Figure 1.** Influence of acetaldehyde concentrations in yeast final biomass (OD 600), in static fermentations. Higher acetaldehyde concentrations (700 to 1000 mg $L^{-1}$) show an inhibitory effect on final yeast biomass. Higher concentrations also show an inhibitory effect on growth rate (**Figure 5A**) and ethanol yield (**Figure 5B**), when compared to lower concentrations. Values above lines represent the exact *p*-value for the specific unpaired *t*-test. Values over lines represent *p*-values from one-sided unpaired *t*-tests. Symbols and error bars represent the mean ± standard deviation. For all the results presented in the figure, n=3 independent experiments. Source data are provided as a Source Data file.

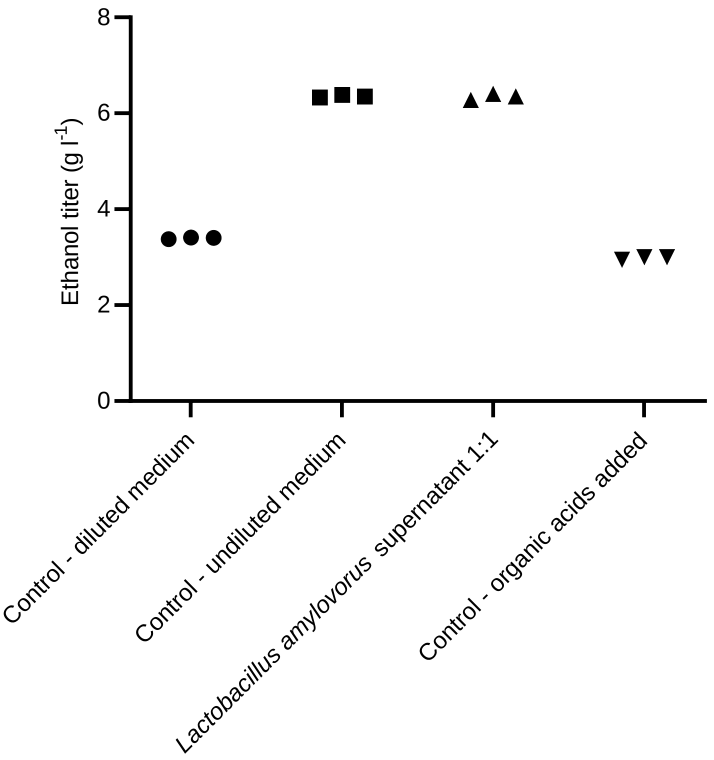


**Supplementary Figure 2.** Final ethanol titre from cross-feeding experiments between *S. cerevisiae* and *L. amylovorus.* Yeast was grown on Milli-Q water diluted SM medium (10x diluted, around 18 g $L^{-1}$ total sugars), herein defined as Control – undiluted medium (black circles). This medium was further diluted (in 1:1 ratio) with either Milli-Q water (Control – diluted medium; black squares); *L. amylovorus* supernatant (*Lactobacillus amylovorus* supernatant 1:1; black triangles); and organic acids solution (Control – organic acids added; inverted black triangles). The organic acids final concentrations were 44.4 and 33.3 for lactic and acetic acid, respectively. Even after the bacterial growth, its supernatant allows yeast to produce as much ethanol as the undiluted control medium.  For all the results presented in the figure, n=3 independent experiments. Source data are provided as a Source Data file.

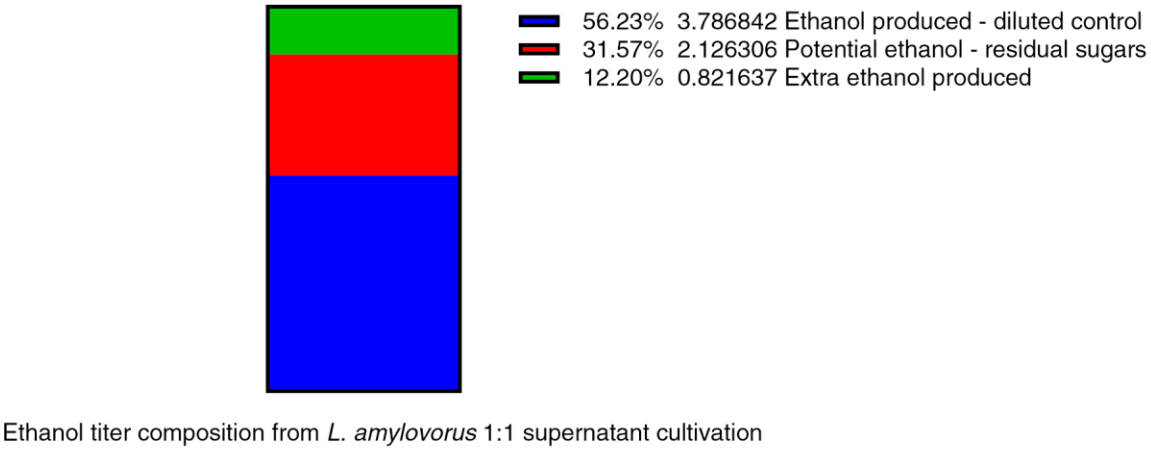


**Supplementary Figure 3.** The hypothesized composition of the ethanol titre from the cross-feeding assays between *S. cerevisiae* and *L. amylovorus*. When cultured in medium diluted with *L. amylovorous* supernatant, the yeast is capable to produce almost as much ethanol as produced in undiluted media. When comparing the ethanol produced in diluted media in Milli-Q water (blue bar) and the maximum theoretical ethanol produced with the residual sugars present in the bacterial supernatant (red bar), there is still some ethanol that was produced, and cannot be directly linked to the available sugars (green bar). This extra ethanol, which represents around 12% of the total ethanol titre in this medium, most likely is originated by the reduction of the available acetaldehyde into ethanol. Source data are provided as a Source Data file.

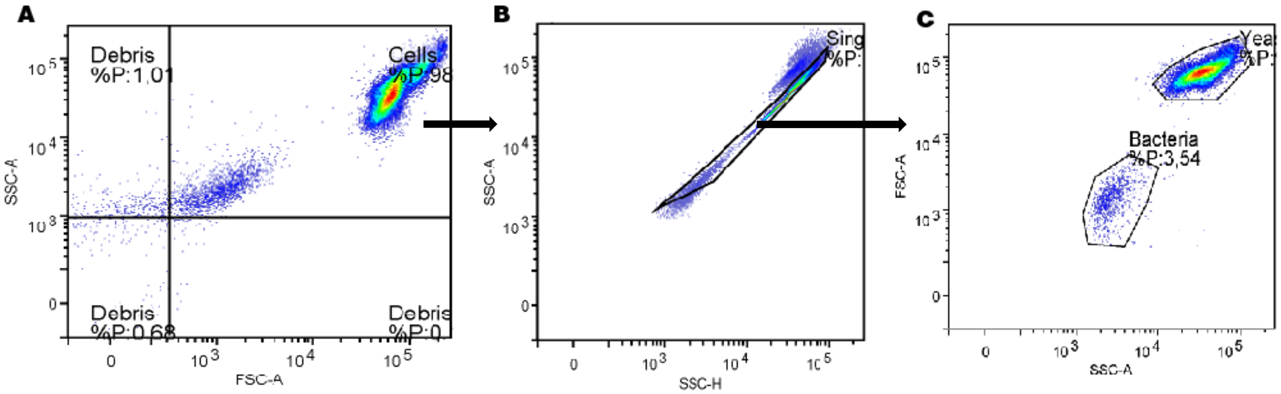


**Supplementary Figure 4.** Representative schematics of the gating strategy applied to resolve both yeast and bacterial populations. **A:** Initially cells are separated from media debris, using side scatter (SSC-A) and front scatter (FSC-A) parameters. The gating named ‘Cells’ contain all the yeast and bacteria cells, and not the media debris. **B:** By comparing area versus height parameters (SSC-A and SSC-H, respectively) from the gated events from the gate ‘Cells’, the singlets are separated from duplets, in the gate ‘Singlets’. **C:** Yeast and bacterial cells are separated based on their volume and granularity, using SSC-A and FSC-A, respectively, from the gate ‘Singlets’.

**Supplementary Table 1.** *p*-values from correlation analysis between microbial species abundance and ethanol yield and yeast cell count.

| | Wilcoxon rank-sum test *p*-values | |
|---|---|---|
| | **Yield ~ species abundance** | **Yeast ~ species abundance** |
| *Lactobacillus amylovorus* | 0.008909926 | 0.043258152 |
| *Lactobacillus fermentum* ST1 | 0.184784135 | 0.308900249 |
| *Lactobacillus fermentum* ST2 | 0.585798644 | 0.508822257 |
| *Lactobacillus buchneri* | 0.614999089 | 0.388508386 |
| *Lactobacillus helveticus* | 0.617095007 | 0.574127815 |
| *Pediococcus claussenii* | 0.645561816 | 0.098938796 |
| *Zymomonas mobilis* | 0.407338789 | 0.248362765 |

**Supplementary Table 2.** Acetaldehyde quantification in bacterial supernatants.

| **Species** | **Acetaldehyde in supernatant (mg $L^{-1}$)** |
|---|---|
| *Lactobacillus amylovorus* | 462.4 |
| *Lactobacillus fermentum* ST1 | 13.1 |
| *Lactobacillus fermentum* ST2 | 5.3 |
| *Lactobacillus buchneri* | 22.6 |
| *Lactobacillus helveticus* | 4.2 |
| *Pediococcus clausenii* | 4.8 |
| *Zymomonas mobilis* | 13.1 |

**Supplementary Table 3.** Metabolite concentration of cross-feeding cultivations between *S. cerevisiae* and *L. amylovorus*. Mean and standard deviation were provided. The experiments were performed in triplicate.

| **Treatment** | **Average concentration (g $L^{-1}$)** | | | | | |
|---|---|---|---|---|---|---|
| | **Glucose** | **Fructose** | **Lactate** | **Acetate** | **Ethanol** | **OD600** |
| Control – Diluted medium | 0.018202 ±0.0021265 | 0.241156 ±0.00249 | 0 | 0 | 3.395046 ±0.013212 | 1.4666667 ±0.018856 |
| Control – Undiluted medium | 0.078059 ±0.0029849 | 0.595608 ±0.001461 | 0 | 0 | 6.353906 ±0.020523 | 4.7866667 ±0.099778 |
| *L. amylovorus* – organic acids added | 0.021737 ±0.0024663 | 0.241885 ±0.006534 | 1.240474 ±0.026823 | 0.919556 ±0.019908 | 6.342989 ±0.050659 | 4.96 ±0.142361 |
| Control – organic acids added | 0.078006 ±0.0045702 | 0.414379 ±0.005271 | 3.0000014 ±0.005049 | 1.927596 ±0.008593 | 2.986566 ±0.025535 | 1.4666667 ±0.018856 |
| *L. amylovorus* supernatant | 1.99206 | 2.168194 | 1.293807 | 0.549656 | 0 | |

**Supplementary Table 4**. Acetaldehyde quantification in bacterial supernatant.

| **Supernatant** | **Average cetaldehyde (mg $L^{-1}$)** | **Standard deviation** |
|---|---|---|
| *L. amylovorus* | 650.4 | 55.8 |

**Supplementary Table 5.** Acetaldehyde quantification in the fermented broth of sugarcane ethanol fermentations contaminated with *L. amylovorus.*

| **Treatment** | **Average acetaldehyde (mg $L^{-1}$)** | **Standard deviation** |
|---|---|---|
| Control – cycle 2 | -0.048735333 | 0.046707189 |
| *L. amylovorus* $10^4$ cells $mL^{-1}$ – cycle 2 | -0.017235667 | 0.032563827 |
| *L. amylovorus* $10^5$ cells $mL^{-1}$ – cycle 2 | -0.030311 | 0.041279434 |
| *L. amylovorus* 106 cells mL-1 – cycle 2 | -0.072508667 | 0.038083737 |
| Control – cycle 3 | 0.005349 | 0.064105401 |
| *L. amylovorus* $10^4$ cells $mL^{-1}$ – cycle 3 | 0.014858333 | 0.035906785 |
| *L. amylovorus* $10^5$ cells $mL^{-1}$ – cycle 3 | -0.046952333 | 0.040823389 |
| *L. amylovorus* $10^6$ cells $mL^{-1}$ – cycle 3 | -0.026150667 | 0.006061035 |
