# Supplementary material for: Complex yeast–bacteria interactions affect the yield of industrial ethanol fermentation
Source: Nat Commun. 2021 Mar 8;12:1498. doi: 10.1038/s41467-021-21844-7 (PMC7940389; doi:10.1038/s41467-021-21844-7)
Supplement: Supplementary file 3 — Reporting Summary [file 41467_2021_21844_MOESM3_ESM.pdf]

nature research

Corresponding author(s): Morten Otto Alexander Sommer

Last updated by author(s): Jan 30, 2021

Reporting Summary

Nature Research wishes to improve the reproducibility of the work that we publish. This form provides structure for consistency and transparency in reporting. For further information on Nature Research policies, see our Editorial Policies and the Editorial Policy Checklist.

Statistics

For all statistical analyses, confirm that the following items are present in the figure legend, table legend, main text, or Methods section.

n/a	Confirmed	
☐	☒	The exact sample size (*n*) for each experimental group/condition, given as a discrete number and unit of measurement
☐	☒	A statement on whether measurements were taken from distinct samples or whether the same sample was measured repeatedly
☐	☒	The statistical test(s) used AND whether they are one- or two-sided<br>*Only common tests should be described solely by name; describe more complex techniques in the Methods section.*
☐	☒	A description of all covariates tested
☐	☒	A description of any assumptions or corrections, such as tests of normality and adjustment for multiple comparisons
☐	☒	A full description of the statistical parameters including central tendency (e.g. means) or other basic estimates (e.g. regression coefficient) AND variation (e.g. standard deviation) or associated estimates of uncertainty (e.g. confidence intervals)
☐	☒	For null hypothesis testing, the test statistic (e.g. *F*, *t*, *r*) with confidence intervals, effect sizes, degrees of freedom and *P* value noted<br>*Give P values as exact values whenever suitable.*
☒	☐	For Bayesian analysis, information on the choice of priors and Markov chain Monte Carlo settings
☒	☐	For hierarchical and complex designs, identification of the appropriate level for tests and full reporting of outcomes
☒	☐	Estimates of effect sizes (e.g. Cohen's *d*, Pearson's *r*), indicating how they were calculated

Our web collection on statistics for biologists contains articles on many of the points above.

Software and code

Policy information about availability of computer code

Data collection	Microsoft Excel 2016; Microsoft Word 2016
Data analysis	Microsoft Excel 2016; Prism 8 and 9; R Version 4.0.3; CarveMe 1.4.1; Python 3.7; COBRApy 0.13.3

For manuscripts utilizing custom algorithms or software that are central to the research but not yet described in published literature, software must be made available to editors and reviewers. We strongly encourage code deposition in a community repository (e.g. GitHub). See the Nature Research guidelines for submitting code & software for further information.

Data

Policy information about availability of data

All manuscripts must include a data availability statement. This statement should provide the following information, where applicable:

- Accession codes, unique identifiers, or web links for publicly available datasets
- A list of figures that have associated raw data
- A description of any restrictions on data availability

Data supporting the findings of this work are available within the paper and its Supplementary Information files. A reporting summary for this Article is available as a Supplementary Information file. The datasets and materials generated and analyzed during the current study are available from the corresponding author upon request. The source data underlying Figures 2a, 2b, 3, 4a, 4b, 5a, 5b, 5c, 6a, 6b, 6c, 6d, 6e and 6f as well as Supplementary Figures 1, 2, and 3 are provided as a Source Data file. In addition, please find the used genomes accession numbers and hyperlinks: The yeast model used was the iMM904 (http://bigg.ucsd.edu/models/iMM904), generated from the genome GCF_000146045.2, from S. cerevisiae strain S288C. The L. amylovorus strain 30SC model was created from genome GCA_000191545.1 (https://www.ncbi.nlm.nih.gov/assembly/GCA_000191545.1).

Field-specific reporting

Please select the one below that is the best fit for your research. If you are not sure, read the appropriate sections before making your selection.

☒ Life sciences ☐ Behavioural & social sciences ☐ Ecological, evolutionary & environmental sciences

For a reference copy of the document with all sections, see nature.com/documents/nr-reporting-summary-flat.pdf

Life sciences study design

All studies must disclose on these points even when the disclosure is negative.

Sample size	Sample size was calculated based on the number of potential combinations with the available species in the study. Triplicates were considered sufficient based on the variability of previous experiments.
Data exclusions	No data were excluded from the analysis.
Replication	The methodology applied in this study has been validated before in published peer-reviewed papers. Each methodology was internally replicated at least in three independent experiments, before its adoption on the experiments described in this study.
Randomization	Randomization was not possible in this study, since the synthetic microbial communities had to be constructed based on every possible consortium composition. In that way, the composition followed this rationale, instead of being assembled randomly. In order to reduce the risk of biases based on experimental design, each condition was performed in triplicate, and each of the replicates position in the microplate was randomly assigned.
Blinding	Bliding during data collection was not possible, since the researcher involved on designing the synthetic communities needed to know which species were being introduced or not in each community composition. During data analysis, internal codes were used instead of species name (e.g. Lactobacillus fermentum strain 1 = sp2st1). In this way, the results produced from data analysis of the microbial communities were unbiased.

Reporting for specific materials, systems and methods

We require information from authors about some types of materials, experimental systems and methods used in many studies. Here, indicate whether each material, system or method listed is relevant to your study. If you are not sure if a list item applies to your research, read the appropriate section before selecting a response.

Materials & experimental systems

n/a	Involved in the study	
☒	☐	Antibodies
☒	☐	Eukaryotic cell lines
☒	☐	Palaeontology and archaeology
☒	☐	Animals and other organisms
☒	☐	Human research participants
☒	☐	Clinical data
☒	☐	Dual use research of concern

Methods

n/a	Involved in the study	
☒	☐	ChIP-seq
☐	☒	Flow cytometry
☒	☐	MRI-based neuroimaging

Flow Cytometry

Plots

Confirm that:

☒ The axis labels state the marker and fluorochrome used (e.g. CD4-FITC).

☒ The axis scales are clearly visible. Include numbers along axes only for bottom left plot of group (a 'group' is an analysis of identical markers).

☒ All plots are contour plots with outliers or pseudocolor plots.

☒ A numerical value for number of cells or percentage (with statistics) is provided.

Methodology

Sample preparation	A sample from each well (10ul) was taken after overnight cultivation, transferred to a new microplate, and diluted in 190 ul of PBS buffer (pH 7.4). Yeast and bacterial populations were resolved via front and side scatter comparison (FSC versus SSC).

Instrument	BD LSRFortessa
Software	FlowJo v10.
Cell population abundance	Yeast and bacteria populations were above 10000 cells/ul. Purity of samples was determined by comparing cells against a blank control , comprised of fermentation media diluted in PBS buffer.
Gating strategy	Singlets were sorted using gates SSC-A (area)/SSC-H (height). After sorted, cells were separated from debris based on previous gating of blank control. Yeast and bacteria populations were sorted using FSC/SSC gates.

☒ Tick this box to confirm that a figure exemplifying the gating strategy is provided in the Supplementary Information.
