# Supplementary material for: Complex yeast–bacteria interactions affect the yield of industrial ethanol fermentation
Source: Nat Commun. 2021 Mar 8;12:1498. doi: 10.1038/s41467-021-21844-7 (PMC7940389; doi:10.1038/s41467-021-21844-7)
Supplement: Supplementary file 2 — Peer Review File [file 41467_2021_21844_MOESM2_ESM.pdf]

Reviewers' comments:

Reviewer #1 (Remarks to the Author):

The paper "Complex yeast-bacteria interactions affect the yield of industrial ethanol fermentations" by de Oliveira Lino et al. is an interesting study on the yeast-bacterial interaction during ethanol production. It may be interesting that the presence of L. amylovorus increase ethanol. However, this paper feels very preliminary. All the studies are done in small volumes in multiwell plates. A fermentation on a small fermenter should be done to confirm that the increase of ethanol is real in conditions closer to industrial conditions, that are very different from the very small volumes on plates, where oxygenation may be very different. The results points to acetaldehyde as the molecule that act as a bridge between bacteria and yeast, but more work has to be included to prove that for real, like mutants of L. amylovorus with lower of higher acetaldehyde that may change ethanol accordingly.

Lactic acid is other bacterial metabolite that may influence glucose repression in yeast (1), and that could change also ethanol yield. Comment this possibility.

1. Garcia DM, Dietrich D, Clardy J, Jarosz DF. A common bacterial metabolite elicits prion-based bypass of glucose repression. Elife [Internet]. 2016;5. Available from: https://www.ncbi.nlm.nih.gov/pubmed/27906649

Reviewer #2 (Remarks to the Author):

The article by Lino, et al. describes interactions between yeast and common bacterial contaminants in small-scale sugarcane ethanol fermentations. They use 96-well block fermentations to reconstitute combinations of yeast with 7 bacterial "contaminants" and measure the chemical output (metabolites via HPLC) and microbial population structure (yeast vs bacteria via flow cytometry). They also assess higher order interactions and attempt to extract functional information from the data. The data point to an intriguing link between a potential industrial probiotic bacterium and increased ethanol yields. Overall the manuscript is well-conceived, experimentally sound, and of high interest to both industrial microbiologists and those interested in understanding the functioning of synthetic microbial communities. Comments below:

- Abstract: The first line of the abstract is a throwaway line. There is also really no need to bring up human health here or elsewhere.
- Line 56: Some will take issue with the phrase "real world" here to describe a microbial community.
- Line 70: is 7-11% w/v?
- Figure 1: The two Lactobacillus fermentum strains are alternately referred to as strains 1 and 2, and strains A and B in this figure.
- Line 120-122: How were these numbers calculated? I'm assuming this is from the flow cytometry data?
- Line 336: de Man Rogosa Sharpe

Reviewer #3 (Remarks to the Author):

Microbial communities are shaped and stabilized by the interactions between their constituent members. In this manuscript, taking the microbial community in sugarcane ethanol fermentations as an example, the authors analyzed the microbial interactions in sugarcane ethanol fermentation by reconstituting different possible combinations and argued that Lactobacillus amylovorus improves the

yeast growth rate and ethanol yield by cross-feeding.

Although the manuscript presents an interesting research topic, I found that the experiments were not well designed and the results that support the author's arguments seem ambiguous. The technical soundness and completeness of the paper is below the bar for publishing in Nature Communications.

Major comments:

1. The authors showed that fermentation time was 24 h. Did the yeast reach the steady state after 24 h during a statistic condition? Why did they extend the fermentation time and showed the time course results of the cell counts and ethanol yields?

2. The fermentation condition was static. Why the rate of yeast growth was analyzed in the presence of different acetaldehyde concentrations under agitation?

3. The argument on acetaldehyde cross-feeding is not convincing. The authors should present an additional experiment that clearly shows the role of L. amylovorus in ethanol and mass production of S. cerevisiae in a larger scale of fermentation, such as a flask or a lab-scale bioreactor fermentation. Additionally, by constructing a synthetic microbial community of L. amylovorus and S. cerevisiae in different ratios, the optimal ratio of two strains can be obtained.

4. To additionally demonstrate the acetaldehyde cross-feeding impacts the ethanol production, authors should also compare the acetaldehyde concentration of the optimal synthetic microbial community with optimal acetaldehyde concentration obtained in Fig. 5.

5. Does the presence of L. amylovorus increase acidity? Lactic acid bacteria usually produce lactic acid, increasing the acidity of whole cultivation and hence placing a stress over the whole community. The viability of S. cerevisiae in the consortium can be impaired if the concentration of L. amylovorus is high. The authors need to quanfiy the lactic acid concentration and acidity of media with or without L. amylovorus.

6. The authors shall conduct a sugarcane fermentation with synthetic microbial community of L. amylovorus and S. cerevisiae with the optimal ratio. If the impact of L. amylovorus is obvious, the ethanol and biomass concentration would be increased in comparison with those of S. cereviaise alone. That result can solidify that synthetic microbial community can be applied to the industrial level of ethanol production using sugarcane ethanol fermentation.

7. Line 32, the authors shall provide an explanation of the relationship of the probiotic and bioethanol at the "Introduction" section. It is unclear why the authors introduce the concept of probiotic.

Reviewers rebuttal

Reviewer #1 (Remarks to the Author):

The paper "Complex yeast-bacteria interactions affect the yield of industrial ethanol fermentations" by de Oliveira Lino et al. is an interesting study on the yeast-bacterial interaction during ethanol production. It may be interesting that the presence of L. amylovorus increase ethanol. However, this paper feels very preliminary. All the studies are done in small volumes in multiwell plates. A fermentation on a small fermenter should be done to confirm that the increase of ethanol is real in conditions closer to industrial conditions, that are very different from the very small volumes on plates, where oxygenation may be very different. The results points to acetaldehyde as the molecule that act as a bridge between bacteria and yeast, but more work has to be included to prove that for real, like mutants of L. amylovorus with lower of higher acetaldehyde that may change ethanol accordingly. Lactic acid is other bacterial metabolite that may influence glucose repression in yeast (1), and that could change also ethanol yield. Comment this possibility.
1. Garcia DM, Dietrich D, Clardy J, Jarosz DF. A common bacterial metabolite elicits prion-based bypass of glucose repression. Elife [Internet]. 2016;5. Available from:https://www.ncbi.nlm.nih.gov/pubmed/27906649

Reply: We would like to thank the reviewer for pointing out the limitations of the original submitted study. We agree that an experiment in larger scale, capable of better simulating the industrial fermentation reality, is an important addition to the study. We have now performed the suggested experiment and included it in the revised version. In this experiment, we simulate as far as possible the Brazilian sugarcane ethanol fermentation – including all its unitary steps – at laboratory scale[1]. In this experiment, we analyze the effect of *L. amylovorus* addition, in difference concentrations (cell ml^{-1}) in the yeast physiology and fermentation performance. We are able to observe that, indeed, *L. amylovorus* addition to the fermentation benefits the industrial process (i.e. higher fermentation yields by almost 3% in the presence of the bacteria), without the expense of jeopardizing the yeast population (i.e. no significant changes in yeast biomass production, viability, etc.).

We also appreciate the reviewer's suggestion on testing the effect on yeast growth and ethanol production of *L. amylovorus* mutants with impaired acetaldehyde production capacity. Although we consider this an interesting approach to answer these questions, we believe that such approach has its own caveats and increases significantly the level of complexity of this study. Since we are considering the effect of cross-feeding among different species, a mutant strain with modifications in its central carbon metabolism would probably result in different titers of other metabolites, which could affect the interaction of both species in different ways. Moreover, it would also be necessary to perform a full genome sequencing of such strains, with high resolution, in order to demonstrate that no off target mutation was present.

We have chosen to answer this question by performing cross-feeding experiments with yeast in media mixed with *L. amylovorus* supernatants. We show that medium supplemented with *L. amylovorus* supernatant results in higher ethanol titers, when compared to media mixed with water. The first logical

explanation is that the residual sugars present in the bacteria supernatant would be the cause of the extra ethanol titer. However, when analyzing the final ethanol titer and the residual sugar content we find that the ethanol titer is almost 12% higher than the theoretical maximum that could be obtained considering the amount of sugars. Intriguingly, this extra ethanol titer is in par with the acetaldehyde concentration present in the supernatant, which again suggests that the cross-feeding among these two species occur via this molecule. This dataset, together with the previous analysis of the effect of acetaldehyde addition in *S. cerevisiae* fermentations, further strengthen our claims.

The reviewer presents a valid point regarding other bacterial metabolites, like lactic acid. We demonstrate in our higher scale fermentations, that although the acidity titers ($g_{organic\ acids}\ l^{-1}$) are significantly higher when *L. amylovorus* is added to the process, no significant changes on yeast physiology can be observed.

Also, when culturing yeast in initially high organic acids titers, for both lactic and acetic acids (44.4 and 33.3 mM, respectively), we observe that there is an impact on final growth of the yeast population, although it was still able to grow. Considering the initial yeast inoculum and organic acids concentration, we observe that the actual organic acids produced by this bacteria in the simulated industrial fermentations are considerably lower, resulting in negligible impacts to yeast metabolism.

Reviewer #2 (Remarks to the Author):

The article by Lino, et al. describes interactions between yeast and common bacterial contaminants in small-scale sugarcane ethanol fermentations. They use 96-well block fermentations to reconstitute combinations of yeast with 7 bacterial "contaminants" and measure the chemical output (metabolites via HPLC) and microbial population structure (yeast vs bacteria via flow cytometry). They also assess higher order interactions and attempt to extract functional information from the data. The data point to an intriguing link between a potential industrial probiotic bacterium and increased ethanol yields. Overall the manuscript is well-conceived, experimentally sound, and of high interest to both industrial microbiologists and those interested in understanding the functioning of synthetic microbial communities. Comments below:

• Abstract: The first line of the abstract is a throwaway line. There is also really no need to bring up human health here or elsewhere.

Reply: We appreciate the reviewer's input and have suppressed this passage deemed as unnecessary.

• Line 56: Some will take issue with the phrase "real world" here to describe a microbial community.

Reply: we understand the reviewer's concern regarding this term, and have changed it accordingly to "actual", which we believe is more suitable to express our thoughts in this phrase.

• Line 70: is 7-11% w/v?

Reply: Indeed, this information was missing. The correct unity is v/v. We would like to thank the reviewer on pointing this missing bit of information. We have included it on this new version of the manuscript.

• Figure 1: The two Lactobacillus fermentum strains are alternately referred to as strains 1 and 2, and strains A and B in this figure.

Reply: We would like to thank the reviewer for observing this issue. We have now normalized these strains nomenclature as strains 1 and 2 throughout the manuscript.

• Line 120-122: How were these numbers calculated? I'm assuming this is from the flow cytometry data?

Reply: Indeed, it was calculate via flow-cytometry. We apologize for not mentioning this on the original manuscript, and have now included the information on the revised version.

• Line 336: de Man Rogosa Sharpe

Reply: We would like to thank the reviewer for pointing this typo on the text. We have correct the name of the media for de Man Rogosa Sharpe now.

Reviewer #3 (Remarks to the Author):

Microbial communities are shaped and stabilized by the interactions between their constituent members. In this manuscript, taking the microbial community in sugarcane ethanol fermentations as an example, the authors analyzed the microbial interactions in sugarcane ethanol fermentation by reconstituting different possible combinations and argued that *Lactobacillus amylovorus* improves the yeast growth rate and ethanol yield by cross-feeding.

Although the manuscript presents an interesting research topic, I found that the experiments were not well designed and the results that support the author's arguments seem ambiguous. The technical soundness and completeness of the paper is below the bar for publishing in Nature Communications.

Major comments:

1. The authors showed that fermentation time was 24 h. Did the yeast reach the steady state after 24 h during a statistic condition? Why did they extend the fermentation time and showed the time course results of the cell counts and ethanol yields?

Reply: We appreciate the reviewer's critique over this experimental set. The yeast population had stopped growing before 24h. We extended the fermentation time to ensure that the fermentations would been sampled on its endpoint, so we could more accurately infer the impact of different conditions in yeast growth and ethanol production.

2. The fermentation condition was static. Why the rate of yeast growth was analyzed in the presence of different acetaldehyde concentrations under agitation?

Reply: We appreciate the reviewer's observation on this matter. This done because yeast cells tend to decant and accumulate on the bottom of the plate wells. This impedes a precise measurement of OD throughout the course of the fermentation. In order to circumvent this issue, we performed such cultivations under agitation, since our main objective with these was to calculate yeast's growth rates.

3. The argument on acetaldehyde cross-feeding is not convincing. The authors should present an additional experiment that clearly shows the role of L. amylovorus in ethanol and mass production of S. cerevisiae in a larger scale of fermentation, such as a flask or a lab-scale bioreactor fermentation. Additionally, by constructing a synthetic microbial community of L. amylovorus and S. cerevisiae in different ratios, the optimal ratio of two strains can be obtained.

Reply: We agree with the reviewer's observations. In order to address these limitations of the original manuscript we have performed a new set of experiments, where we simulate, as far as possible, the Brazilian sugarcane ethanol production process at laboratory scale[1]. With this setup, we were able to analyze the influence of *L. amylovorus* addition in yeast physiology and fermentation performance. We have also analyzed different concentrations of *L. amylovorus* inoculum, and found that even on the lower end of the inoculum concentration (i.e. 10^4 cells ml^{-1}) it is possible to observe significant differences on the final ethanol yield values.

4. To additionally demonstrate the acetaldehyde cross-feeding impacts the ethanol production, authors should also compare the acetaldehyde concentration of the optimal synthetic microbial community with optimal acetaldehyde concentration obtained in Fig. 5.

Reply: We thank the reviewer for this suggestion. We have indeed performed this analysis but, unfortunately, it is not possible to observe a significant difference on the final titer of acetaldehyde in co-cultures, since the yeast population – which is larger than its bacterial counterpart – is actively consuming the acetaldehyde released in the medium. The final concentration of the residual acetaldehyde was similar to all analyzed conditions, suggesting that yeast indeed consumes it (since it was possible to observe differences on the ethanol yield among different populations).

5. Does the presence of L. amylovorus increase acidity? Lactic acid bacteria usually produce lactic acid, increasing the acidity of whole cultivation and hence placing a stress over the whole community. The viability of S. cerevisiae in the consortium can be impaired if the concentration of L. amylovorus is high.

The authors need to quanfiy the lactic acid concentration and acidity of media with or without L. amylovorus.

Reply: We appreciate the reviewer's comment and suggestion. Indeed, the organic acids titer (g l^{-1}) in fermentations where *L. amylovorus* was added were higher than control fermentations, where only the yeast population was present. However, the higher organic acids titer did not impair yeast fermentation, nor it had any effect on yeast viability. Moreover, we have also conducted a small-scale experiment, where we grow yeast, under high initial organic acids concentration for both lactic and acetic acids (44.4 and 33.3 mM, respectively). In this assay, it is possible to observe that the yeast growth was impacted, but it was still able to grow. In actual fermentations, where the yeast initial population is considerably higher (i.e. *ca.* 10^8 cells ml^{-1}) the impact of the organic acids produced by *L. amylovorus* population in yeast physiology was negligible.

6. The authors shall conduct a sugarcane fermentation with synthetic microbial community of L. amylovorus and S. cerevisiae with the optimal ratio. If the impact of L. amylovorus is obvious, the ethanol and biomass concentration would be increased in comparison with those of S. cereviaise alone. That result can solidify that synthetic microbial community can be applied to the industrial level of ethanol production using sugarcane ethanol fermentation.

Reply: We thank the reviewer's suggestion. As answered on question 3, we have included this experiment in the revised version of the manuscript. Indeed, it is possible to observe an increase in ethanol yield, of almost 3%, when *L. amylovorus* is inoculated in the fermentation. This increase in yield occurs without any observable negative impact on the yeast population, an important matter for a process that recycles its yeast biomass through the course of the crop season. We believe that this new dataset strengthens our previous arguments as applying the concept of synthetic microbial communities in industrial fermentations.

7. Line 32, the authors shall provide an explanation of the relationship of the probiotic and bioethanol at the "Introduction" section. It is unclear why the authors introduce the concept of probiotic.

Reply: We appreciate the reviewer's comments and have altered the text in this section, providing more clarity to this used term.

Cited references in the rebuttal

1. Raghavendran, V., Basso, T. P., da Silva, J. B., Basso, L. C. & Gombert, A. K. A simple scaled down system to mimic the industrial production of first generation fuel ethanol in Brazil. *Antonie Van Leeuwenhoek* **110**, 971–983 (2017).

Reviewer #1 (Remarks to the Author):

The paper is interesting, but to be fit for a high profile journal it has to be proven that co-cultivation produces significant increase of ethanol in industrial conditions AND to have a molecular explanation of the process. That would imply using mutants of Lactobacillus amylovorus as I suggested in the first review.
The main aim of the paper is to study the ecology of mixed fermentations, and that goal is reached, but that is not of general interest to justify publication.

Minos point: do not refer to the growth media as beer or wine.

Reviewer #2 (Remarks to the Author):

Critiques from my previous reviews have been sufficiently addressed. The additional experimental work strengthens the manuscript.

Reviewer #3 (Remarks to the Author):

The authors have addressed my previous concerns in their revised paper. I recommend publication for this manuscript.

Reviewer #1 (Remarks to the Author):

The paper is interesting, but to be fit for a high profile journal it has to be proven that co-cultivation produces significant increase of ethanol in industrial conditions AND to have a molecular explanation of the process. That would imply using mutants of Lactobacillus amylovorus as I suggested in the first review.

The main aim of the paper is to study the ecology of mixed fermentations, and that goal is reached, but that is not of general interest to justify publication.

Minos point: do not refer to the growth media as beer or wine.

Reply: We have avoided the terms "beer" and "wine" in the revision.

Reviewer #2 (Remarks to the Author):

Critiques from my previous reviews have been sufficiently addressed. The additional experimental work strengthens the manuscript.

Reviewer #3 (Remarks to the Author):

The authors have addressed my previous concerns in their revised paper. I recommend publication for this manuscript.
